# Autologous Hematopoietic Stem Cell Transplantation for Multiple Myeloma without Cryopreservation

**DOI:** 10.1155/2012/917361

**Published:** 2012-05-28

**Authors:** Khalid Ahmed Al-Anazi

**Affiliations:** Section of Adult Hematology and Hematopoietic Stem Cell Transplantation, Oncology Center, King Fahad Specialist Hospital, P.O. Box 15215, Dammam 31444, Saudi Arabia

## Abstract

High-dose chemotherapy followed by autologous hematopoietic stem cell transplantation is considered the standard of care for multiple myeloma patients who are eligible for transplantation. The process of autografting comprises the following steps: control of the primary disease by using a certain induction therapeutic protocol, mobilization of stem cells, collection of mobilized stem cells by apheresis, cryopreservation of the apheresis product, administration of high-dose pretransplant conditioning therapy, and finally infusion of the cryopreserved stem cells after thawing. However, in cancer centers that treat patients with multiple myeloma and have transplantation capabilities but lack or are in the process of acquiring cryopreservation facilities, alternatively noncryopreserved autologous stem cell therapy has been performed with remarkable success as the pretransplant conditioning therapy is usually brief.

## 1. Introduction

Multiple myeloma (MM) accounts for 1% of all cancers and about 10% of all hematologic malignancies [1]. It is characterized by neoplastic proliferation of a clone of plasma cells producing a monoclonal immunoglobulin and can present as a single lesion (plasmacytoma) or multiple lesions (MM). Clonal plasma cells proliferate in the bone marrow and can cause extensive lytic bony lesions, osteopenia, and pathological fractures [2]. MM is a heterogenous disease rather than a single disease entity, as some patients progress rapidly despite therapy, whilst others may not require active therapy for a number of years [2].

Once the diagnosis of MM is made, the patient undergoes staging evaluation in order to start an appropriate line of therapy. The international staging system (ISS) divides patients into 3 categories according to serum albumin and beta-2-microglobulin levels. Conventional cytogenetics, fluorescence *in situ* hybridization (FISH), and molecular studies help to stratify patients into standard-risk, high-risk, and ultra-high-risk groups to determine prognosis and to refine management of patients. Gene expression profiling and plasma cell labeling index can identify high-risk groups and select the most appropriate novel therapies to be used [1–6].

## 2. Use of Novel Agents

The availability of novel agents has expanded treatment options and has improved outcomes of myeloma patients. A number of phase III clinical trials have demonstrated the efficacy of novel agent combinations and their superiority to VAD (vincristine, doxorubicin, and dexamethasone) regimen [7, 8]. Some novel agents appear to be active in high-risk patients, for example, those with adverse cytogenetics and molecular markers or certain comorbidities such as renal failure. Characterization of molecular events at cellular and marrow microenvironment levels has provided a platform for the development of various novel drugs in MM including proteasome inhibitors, immunomodulatory drugs, and HDAC (histone deacetylase) inhibitors [7, 8].

Bortezomib (velcade), the first-in-class proteasome inhibitor, was initially approved for the treatment of relapsed/refractory MM as a single agent [9, 10]. However, the great beneficial role it had exhibited in several clinical studies allowed the expansion of its role to become not only an integral part of induction therapy for newly diagnosed MM, but also a valuable element of consolidation and maintenance therapies in the pre- and posttransplant settings [9–15]. Bortezomib and dexamethasone combination has become an important part of standard induction therapy for newly diagnosed myeloma. This combination can be given twice or once weekly. The once-weekly schedule has proven to be equally effective and safer than the twice-weekly regimen specifically for patients more than 65 years of age. Bortezomib can also be safely given in various combinations with other agents including melphalan, cyclophosphamide, thalidomide, doxorubicin, and lenalidomide [7, 9, 11, 13–16]. Despite its safety profile, which allowed use in patients with renal failure and elderly individuals, the following adverse events have been reported: peripheral neuropathy, extramedullary plasmacytomas, gastrointestinal upset, myelotoxicity, and severe pulmonary complications [9, 12, 13, 15–17].

## 3. Autologous Stem Cell Transplantation

Since the mid-1990s, high-dose chemotherapy followed by autologous hematopoietic stem cell transplantation (auto-HSCT) has been considered the standard of care for frontline therapy in MM patients who are eligible for transplantation [18]. The choice of induction therapy has moved from conventional chemotherapy, for example VAD protocol, to newer regimens that incorporate novel agents like thalidomide, lenalidomide, and bortezomib. Upfront use of these agents, with 3-drug combinations in particular, has produced unprecedented rates of complete response (CR) that were never seen with old conventional chemotherapy and subsequent auto-HSCT [19]. Auto-HSCT offered after novel-agent-based induction therapies provides further improvement in the depth of response which is translated into longer progression-free survival, and potentially overall survival [18, 19]. Therefore, novel agents and auto-HSCT are complementary therapeutic strategies in patients with MM [19]. Improving the outcomes of HSCT in the future will require the exploration of novel strategies aimed at addressing the following issues: reduction of morbidity attributed to high-dose therapy, improving the efficacy of conditioning therapies, and the use of novel agents in the post-HSCT period [20].

For transplant-eligible patients, a bortezomib-based induction therapy is associated with improved disease control after HSCT and should, therefore, be considered the standard of care [20]. Moreover, a number of studies incorporating bortezomib as part of induction therapy have shown no adverse impact of bortezomib therapy on the yield of stem cell harvest and engraftment in patients with MM proceeding to transplant [21]. Auto-HSCT is safe and effective, but the outcome is independent of age, time from diagnosis, previous treatment, and conditioning therapy. However, achievement of CR and low international prognostic index at transplant is essential prognostically [22, 23]. High CD34+ stem cell dose correlates well with early hematopoietic reconstitution and improvement of overall survival [22, 24]. Auto-HSCT for patients with MM can be entirely performed at the outpatient department in cancer centers that are fully equipped and can handle any evolving crisis or emergency. Outpatient auto-HSCT can result in shorter hospital stays and low transplant-related mortality and costs [25]. Studies have also shown that the use of certain conditioning therapies for HSCT can result in significant reduction or even abolition of transfusion of blood products, for example, packed red cells and platelets [26].

## 4. Stem Cell Mobilization

Mobilization of stem cells prior to stem cell collection and auto-HSCT in patients with MM is generally composed of 2 parts: the first part comprises the use of certain chemotherapeutic agents that include a single agent like cyclophosphamide or multiple agents in various combinations, with different dose schedules such as VAD, CD (cyclophosphamide and dexamethasone), CAD (cyclophosphamide, adriamycin, and dexamethasone), IVE (ifosfamide, etoposide, and epirubicin), EDAP (etoposide, dexamethasone, cytosine arabinoside, and cisplatin), CDVP (cyclophosphamide, doxorubicin, vincristine and prednisone), and VTD-PACE (bortezomib, thalidomide, dexamethasone, cisplatin, doxorubicin, cyclophosphamide, and etoposide), and the second part is composed of administration of growth factors such as granulocyte colony-stimulating factor (filgrastim; G-CSF), pegylated G-CSF, and plerixafor (Mozobil) in case of poor mobilization [27–40]. Various dose schedules were used in both single- or multiple-agent chemotherapeutic protocols, for example, doses of cyclophosphamide ranged from 1.0 to 7.0 gram/m^2^ [34–38, 40–42]. However, recent studies have shown that adequate numbers of peripheral blood stem cells can be collected using growth factors alone, without prior chemotherapy, and that the use of cyclophosphamide for stem cell mobilization can overcome the suppressive effect of drugs, used in the treatment of MM, like lenalidomide on stem cell collection [41, 42].

Although filgrastim can be used alone for stem cell mobilization, studies have shown that the yield of stem cells was higher in patients mobilized with cyclophosphamide and G-CSF rather than with G-CSF alone, and that under certain circumstances, some regimens may be preferred to others, for example, VAD chemotherapy protocol followed by standard doses of G-CSF has been shown to be as effective as high-dose cyclophosphamide, in addition to being less toxic and allowing outpatient management with reduced cost [28, 31, 34, 35, 41, 42]. G-CSF and pegylated G-CSF may cause severe pain syndromes, and splenic rupture and may even precipitate veno-occlusive crises in patients with sickle cell anemia. However, in patients with sickle cell trait, stem cell mobilization using G-CSF is generally safe. Due to concerns of more serious adverse effects of G-CSF in patients with sickle cell trait, close monitoring of such patients should be maintained [43–47]. Plerixafor, a novel CXCR4 inhibitor, is effective in mobilization of peripheral blood stem cells in myeloma patients who fail conventional mobilization techniques. It has shown good tolerance and high success rates in patients who are labeled as poor mobilizers [29, 30, 32, 33].

## 5. Stem Cell Collection

Once the CD34+ cell count in peripheral blood exceeds 10.0 to 20.0 × 10^6^/kg body weight, stem cell collection by leukapheresis is usually commenced. Most transplant centers make plans to obtain a target of 3.0 to 4.0 × 10^6^ CD34+ cells/kg in case a single auto-HSCT is desired and a target of 6.0 to 8.0 × 10^6^ CD34+ cells/kg in case a tandem transplant is planned [48–50]. The optimal count of CD34+ cells necessary for hematologic reconstitution is not well characterized, but the minimal count of 2.0 to 3.0 × 10^6^ CD34+ cells/kg is generally accepted as the limit required to ensure short- as well as long-term hematologic reconstitution in the majority of patients [31, 40, 42, 48–50]. The yield of stem cell collection depends on a number of factors including age and performance status of the patient, presence of comorbidities, the previous lines of therapy given to the patient, the bone marrow reserve, upfront versus delayed auto-HSCT, the mobilization protocol used, and the technology applied in stem cell collection [42, 49].

## 6. Cryopreservation of Stem Cells

Cryopreservation of hematopoietic stem cells (HSCs) is routinely employed in auto-HSCT setting and is critical for cord blood transplantation. A variety of cryopreservatives have been used with different freezing and thawing techniques used in various transplantation centers. The standard and the most commonly used cryopreservative is DMSO (dimethylsulfoxide) which prevents freezing damage to living cells. DMSO is usually used at concentrations of 10% combined with normal saline and serum albumin. It is generally safe and nontoxic, but clinically it is associated with significant side effects that include nausea, vomiting, and abdominal cramps in addition to cardiovascular, neurological, respiratory, renal, hepatic, and hemolytic adverse effects. Standardization of stem cell processing using cryopreservation or mechanical freezing is of vital importance [51–53]. After cryopreservation and thawing of stem cells, a significant proportion of collected stem cells (20–30%) becomes nonviable due to early irreversible apoptosis. Therefore, systemic control for the viability of CD34+ cells immediately before reinfusion is recommended [54].

## 7. Autologous Transplantation without Cryopreservation

Studies have shown that peripheral blood stem cells (PBSCs) can be stored safely at 4°C for at least 5 days, while the patient receives high-dose chemotherapy. Viability of stem cells decreases progressively from day 5 onwards [55]. Liquid storage of harvested HSCs, either at room temperature or in standard blood refrigerators, is an alternative to cryopreservation. Preclinical data supporting the use of noncryopreserved HSCs are available since 1957. Studies on mice reported successful rescue after administration of lethal doses of total body irradiation and reinfusion of bone marrow cells that had been stored for 11 days at 25°C. Subsequent *in vitro* and clinical studies on humans showed that bone marrow cells can be preserved in liquid state for 2 to 9 days without significant loss of granulocyte/macrophage-committed progenitor cells providing hematologic reconstitution to patients receiving myeloablative therapy [56]. The technique may be of value in 2 scenarios: (1) use in medical institutions from areas with limited economic resources, that is, having infrastructure to treat hematologic malignancies but not cryopreservation facilities and (2) use in medical institutions treating hematologic malignancies and in the process of establishing an HSCT program that will eventually have cryopreservation capabilities [56–59].

The use of noncryopreserved stem cells in transplantation has the following advantages: (1) simplicity of implementation and allowing auto-HSCT to be done entirely as outpatient, (2) reduction of transplant costs, (3) expansion of the number of medical institutions that offer stem cell therapy, (4) prevention of DMSO toxicity, (5) saving time between the last induction therapy and high-dose therapy, and (6) no significant reduction in viability of collected stem cells provided infusion is done within 5 days of collection. On the other hand, noncryopreserved HSCT has the following disadvantages: (1) limitation of the use of standard high-dose schedules employed in auto-HSCT, (2) plenty of coordination between various teams is required regarding timing of stem cell mobilization, apheresis, administration of high-dose therapy, and stem cell transfusion, and (3) inability to store part of the collection and reserving it for second transplant or other purposes in case a rich product is obtained [56–62].

Melphalan, which is the standard chemotherapeutic agent used in conditioning therapy prior to auto-HSCT in MM, becomes undetectable in plasma and urine 1 and 6 hours, respectively, following intravenous infusion of a high dose. Noncryopreserved stem cells can be reinfused as early as 8 hours after high-dose melphalan. Stem cell transfusion 8 to 24 hours following IV administration of melphalan has been reported to be associated with successful grafts. The dose of melphalan can range between 140 and 220 mg/m^2^ [56, 59]. In a systemic review of the published studies on noncryopreserved autologous PBSCT in a variety of malignant hematological disorders including MM, the following results were obtained: (1) median time to neutrophil recovery ranged between 9 and 14 days, (2) median time to platelet recovery ranged between 13.5 and 25 days, and (3) hematopoietic reconstitution was universal in all the studies that included 560 patients. Only 1 graft failure was reported, and it was attributed to an inadequate stem cell dose [56]. Other studies reported neutrophil recovery as late as 27 days and platelet recovery as late as 37 days [57]. Treatment-related mortality was reported to range from 0.0 to 13.0%. The deaths reported were due to infections, heart failure, interstitial pneumonitis, and hepatic veno-occlusive disease [56–59, 61]. As stem cells can be stored without cryopreservation for a limited period of time, conditioning therapies for malignant hematological disorders that require administration over 6 days or more should either be excluded or these conditioning therapies should be changed altogether to be administered over 1 to 3 days. The rule of the sooner the better should therefore be applied so that harvested stem cells should be reinfused within 5 days of collection [56]. Studies comparing overnight storage of autologous stem cell apheresis products at 4°C with immediately cryopreserved products showed no statistically significant difference between the two groups regarding viability of collected stem cells, neutrophil and platelet engraftment days, safety, and even long-term outcome of the primary disease. Additional benefits of overnight storage of harvested products were reduction in costs and processing time [63–65].

## 8. Simplified Cryopreservation Techniques

Simplified methods of cryopreservation, that is, storage of harvested stem cells in mechanical freezers at −80°C using cryoprotective solutions that contain DMSO, have been successfully utilized in various parts of the world. Results of these simplified and less expensive cryopreservation procedures with regard to hematopoietic recovery after myeloablative therapy are comparable to standard cryopreservation techniques [66–70]. For short-term (less than 168 hours) storage of stem cells, the use of a storage medium composed of combination of super cooling, and University of Wisconsin solution was successfully used. Preservation of stem cells beyond 168 hours was associated with reduced viability of stored stem cells [71].

## 9. Tandem Transplantation in Myeloma

In selected subgroups of patients, tandem or second transplants may be more effective than single rounds of high-dose therapy and auto-HSCT. The timely application of a tandem transplant has extended event-free and overall survival independent of the cytogenetics and beta-2-microglobulin in some patients [72]. In most instances, a second auto-HSCT is performed using cryopreserved stem cells collected prior to the first auto-HSCT [49]. The indications for a tandem transplant in myeloma patients include relapse after first auto-HSCT or following prolonged remission and not achieving CR or near CR with the first auto-HSCT. However, in patients who are in CR or near CR, the second auto-HSCT could be performed as a salvage therapy in the future rather than an elective tandem procedure [49, 50, 72, 73].

## 10. Engraftment Syndrome and/or Autologous GVHD

During neutrophilic recovery following HSCT, a constellation of clinical manifestations that include fever, erythematous skin rash, nausea, vomiting, diarrhea, and noncardiogenic pulmonary edema may occur [74]. These clinical features are usually referred to as engraftment syndrome which may be a manifestation of graft versus host reaction. This syndrome reflects cellular and cytokine interactions and may be associated with significant transplant-related mortality and morbidity due to pulmonary leak syndrome and multiorgan failure [74–77]. It has been well reported in autologous HSCT setting, and the extreme form is usually referred to as an autologous form of graft versus host disease (GVHD). The predisposing factors for auto-GVHD include MM as the primary disease, second auto-HSCT, heavily pretreated patients, high CD34+ cells infused, and achievement of high levels of absolute lymphocyte counts after HSCT [74–79]. Early recognition of this syndrome is vital in order to administer appropriate GVHD therapy which includes high-dose corticosteroids, alemtuzumab, infliximab, daclizumab, and etanercept [74–78].

## 11. Conclusion

Auto-HSCT without cryopreservation is feasible and can be performed successfully in cancer centers that have specific skills as well as standardized CD34+ cytometry technique in order to obtain accurate counting of progenitor cells but lack or are in the process of having cryopreservation facilities. It is simple, safe, and cost-effective. However, proper planning and coordination between various teams is vital for efficient mobilization and collection of hematopoietic progenitor cells, administration of the high-dose chemotherapy, and infusion of fresh stem cell products in a timely manner for optimal transplant outcome. 

Managing teams should cautiously use filgrastim in patients with sickle cell disorders and should take into consideration the possible evolution of an engraftment syndrome after a successful autograft.
